# Regulation of PDGFRα^+^ cells and ICC in progesterone-mediated slow colon transit in pregnant mice

**DOI:** 10.1016/j.heliyon.2024.e25227

**Published:** 2024-01-28

**Authors:** Chen Lu, Hui Luo, Ye Wang, Shuang Jing, Jun Zhao, Kexin Zou, Fan Wu, Hao Ying

**Affiliations:** a Department of Obstetrics and Gynecology, Shanghai First Maternity and Infant Hospital, School of Medicine, Tongji University, Shanghai, 200092, China; b Hospital and Institute of Obstetrics and Gynecology, Shanghai Medical College, Fudan University, Shanghai, 200032, China; c Department of Obstetrics and Gynecology, International Peace Maternity and Child Health Hospital, School of Medicine, Shanghai Jiaotong University, Shanghai 200030, China

**Keywords:** Progesterone, PDGFRα^+^ cell, ICC, Colon slow transit, Smooth muscle

## Abstract

**Background:**

Progesterone can inhibit intestinal smooth muscle contraction; however, the specific mechanism remains unclear. Besides smooth muscle cells, smooth muscle has two important mesenchymal cells, namely interstitial cells of Cajal (ICC) and PDGFRα^+^ cells, which induce the contraction and relaxation of smooth muscles. We aimed to explore the regulation of PDGFRα^+^ cells and ICC in progesterone-mediated colon slow transit in pregnant mice.

**Methods:**

Colon transit experiments were performed *in vivo* and *in vitro* to observe slow colon transit. The expression of PDGFRα and c-KIT was detected by Western blot, RT-PCR, and immunofluorescence. An isometric tension experiment was performed to investigate smooth muscle contractions.

**Results:**

The colon transit time in pregnant mice was longer than that in non-pregnant mice. Progesterone significantly blocks colonic smooth muscle contractions. However, when the relaxation and contraction of PDGFRα^+^ cells and ICC are blocked, progesterone cannot inhibit smooth muscle contraction. When the function of only PDGFRα^+^ cells are blocked, progesterone has a more obvious inhibitory effect on smooth muscle in the non-pregnant group than that in the pregnant group. However, when ICC alone was blocked, progesterone inhibited smooth muscle contractions more clearly in pregnant mice. The protein and mRNA expression of PDGFRα was higher and c-KIT was lower in pregnant mice. PDGFRα^+^ cells and ICC from smooth muscle all co-localize progesterone receptors.

**Conclusions:**

Under the regulation of progesterone, the relaxation function of PDGFRα^+^ cells is enhanced and the contraction function of ICC is weakened, leading to the slow colon transit of pregnant mice.

## 1 Introduction

Gastrointestinal motility disorders are the most common clinical complications in pregnant women, and female sex hormones can cause or contribute to symptoms such as heartburn, nausea, vomiting, and constipation [[1], [2], [3]]. Current studies suggest that gastrointestinal motility disorders are caused primarily by increased hormonal changes, especially progesterone, and not by the physical effects of uterine enlargement, as once believed [4,5]. Given the large number of pregnancies complicated by gastrointestinal motility disorders, understanding the physiological and pathological alterations in gastrointestinal motility during pregnancy is essential to provide therapeutic options for pregnant patients with gastrointestinal disorders.

Progesterone is a steroidal hormone secreted by the ovaries that plays a dominant role in maintaining pregnancy and reproductive function [6,7]. The physiological effects of progesterone are mediated by binding to specific intracellular progesterone receptors (PRs) [[8], [9], [10]]. The PR, a member of the nuclear receptor superfamily, is the transcriptional product of structure-related genes [7,9]. Progesterone becomes active by binding to its specific PR and then changes the expression of the target gene [7]. Activated progesterone can inhibit the transmembrane flow of Ca^2+^, promote the release of intracellular Ca^2+^ and induce hyperpolarization of smooth muscle cells, thereby causing a smooth muscle relaxation response [[11], [12], [13]].

Smooth muscles are the main source of motility in gastrointestinal transit; therefore, we believe that gastrointestinal motility disorders during pregnancy may be closely related to functional changes in smooth muscles. In recent years, researchers have proposed that smooth muscles act as a motor unit with SIP syncytium, which includes not only smooth muscle cells (SMC), but also ICC and PDGFRα^+^ cells [[14], [15], [16], [17]]. Among SIP syncytium, ICC-SMC mediates the contraction of smooth muscle, while PDGFRα^+^ cell-SMC mediates the relaxation of smooth muscle, that is, SMC passively contracts or relaxes under the regulation of ICC and PDGFRα^+^ cells. In recent years, scientists have identified a very important functional protein, calcium-activated chloride channel (ANO1), distributed on the ICC, which is also a key ion channel for the ICC to generate pacing currents [[18], [19], [20]]. When the pacing current generated by the ANO1 channel increases to a certain extent, the ICC depolarizes and drives the depolarization of the SMC through gap junctions, thus causing a contractile reaction of the smooth muscle [21]. PDGFRα^+^ cells have recently received the same attention as the ICC. The SK3 channel, a small conductance calcium-activated potassium channel (SK3), was found to be distributed in PDGFRα^+^ cells. When the SK3 channel is activated by inhibitory neurotransmitters it causes intracellular K^+^ outflow, and PDGFRα^+^ cells undergo hyperpolarization [22]. The hyperpolarization response of the SMC is induced through gap junctions to inhibit the activation of L-type Ca^2+^ channels, causing a smooth muscle relaxation response [15,16,22].

Recent studies have found that PRs are expressed on the ICC and PDGFRα^+^ cells of the fallopian tube smooth muscle and are involved in the regulation of tubal peristalsis acting as sensors by integrating the major pacemaker function with signaling mechanisms [[23], [24], [25]]. Based on these findings, we suspected that progesterone may be involved in the regulation of gastrointestinal motility disorders. We aimed to explore the regulation of PDGFRα^+^ cells and the ICC in progesterone-mediated slow colon transit in pregnant mice.

## 2 Materials and methods

### Ethical statement

All animals were obtained from the Experimental Animal Center of Shanghai Tong Ji University School of Medicine. This research complied with the Guide for the Care and Use of Laboratory Animals of the Science and Technology Commission of China (STCC Publication No. 2, revised in 1988). The study protocol was approved by the Committee on the Ethics of Animal Experiments (Permit Number: Hu 686–2009). All operations were performed under anesthesia, and every manipulation of the experimental animals was performed to maximally relieve suffering.

### 2.1 Animals

The animal model used in this study was the C57BL/6J mouse. The control group consisted of 10-week-old non-pregnant female mice. The experimental group consisted of mice that were 16–18 d pregnant and the same age as the control group. There were 7–9 animals in each group. All mice were housed at 24 °C under a 12-h light/dark cycle with free access to water and food.

### 2.2 Colon transit experiment *in vitro*

Before the experiment, animals were fasted for 12 h and allowed free access to water. The mice were sacrificed the next day, the colon tissue was removed, and feces were cleared using the water pressure of a syringe. The colon specimen was placed in a 37 °C pre-oxygenated (95 % O2:5 % CO2) Krebs solution for 30 min to restore the contractile activity of the colon. Subsequently, experiments were initiated. First, a fecal-sized bead was placed at the proximal end of the colon. A digital camera was used to record the time taken by the beads to travel from the proximal to distal colon. Finally, the transmission speed of the colon was calculated according to the length of the colon divided by the transit time.

### 2.3 Colon/Whole digestive tract transit experiment *in vivo*

The mice were fasted for 12 h and provided free access to water prior to the experiment. The next day, the mice were anesthetized and a hollow bead the size of feces was inserted into a glass tube with a diameter of 3 mm and fixed at one end of the glass tube. Finally, the bead was pushed through the glass tube from the anus of the mouse towards the rectum and proximal colon. Notably, the beads pushed approximately 10 cm from the proximal colon of pregnant mice and 8 cm from the proximal colon of non-pregnant mice. When the bead was pushed into the proximal colon, the glass tube was extracted and the timing began. Finally, we observed the time taken for the mice to expel the beads from the anus.

The transit time of the entire digestive tract of the mice was measured by gavage with carmine solution. First, the mice were moderately anesthetized, and then the left hand held the fur on the back of the mice such that the head, neck, and abdomen of the mice were in a straight line, which allowed the gavage device into the stomach of the mice. Next, the gavage device was connected to a syringe containing the 0.5 mL carmine solution. Finally, the gavage device was inserted approximately 2–3 cm from the mouth into the stomach of the mice, and carmine solution was injected. The timing began when the mice awoke from the anesthesia and ended when the mouse expelled the first red bead from the anus. The transit time of the entire digestive tract was recorded.

### 2.4 Colonic migrating motor complex (CMMC) experiment

Mice were sacrificed by cervical dislocation under general anesthesia. Then, the abdomen was opened along the ventral midline, the colon was removed and quickly placed into a 37 °C pre-oxygenated (95 % O2:5 % CO2) Krebs solution. The mesentery at each end of the colon was fixed using thin steel needles. A syringe was used to inject water into one end of the colon to artificially discharge all feces with water pressure; this procedure must be performed with caution. Next, a glass tube with a fecal-sized bead at one end was inserted along the longitudinal axis into the lumen of the colon. The colon specimen was gently perfused with Krebs solution and left for 40 min to recover colonic contraction activity. A silk thread (USP 5/0) was attached to both ends of the colon and a tension of 0.1 g was applied to the empty colon. The transit wave of the colon was recorded by using an isometric force transducer (RM6240C; Chengdu Instrument Factory, China) linked to an amplifier.

### 2.6 Preparation of smooth muscle tissue & isometric tension measurement of the smooth muscle contraction experiment

Colons were obtained as described above. The colon was cut along the longitudinal axis, feces were removed, and the colon was pinned onto a Sylgard dish with the mucosa facing upward. The mucosa and submucosa were carefully removed under a dissecting microscope and the smooth muscle tissue was exposed. Smooth muscle strips (approximately 2 × 8 mm) were obtained by cutting along the circular axis of the smooth muscle tissue. A silk thread (USP 5/0) was attached to both ends of the muscle strips and attached along the circular axis into 10 mL organ baths containing 37 °C pre-oxygenated (95 % O2:5 % CO2) Krebs solution. Tension (0.2 g) was applied to the muscle strip, which was equilibrated for at least 40 min before recovery of contraction activity. The recording device was identical to that used in the CMMC experiments described above.

### 2.7 Western blot analysis

Proteins were extracted from colon smooth muscle tissues. Samples were lysed in a radioimmunoprecipitation assay buffer (1:100; Beyotime Chemical Co., Jiangsu, China) and PMSF buffer (Beyotime Chemical Co.) containing a protease inhibitor cocktail (Beyotime Chemical Co.). The suspension was centrifuged at 12,000 rotations per minute (rpm) for 15 min at 4 °C. Protein samples (40 μg/lane) were separated on a 10 % sodium dodecyl sulfate polyacrylamide gel and transferred from the polyacrylamide gel to a polyvinylidene difluoride membrane (Bio-Rad Laboratories, Inc., Hercules, CA, USA). After blocking was performed with 5 % non-fat milk in 0.1 % Tris-buffered saline/Tween 20, then the primary antibodies were incubated in polyvinylidenedifluoride membranes overnight at 4 °C. After washing thrice with Tris-buffered saline containing Tween-20 (0.05 %), horseradish peroxidase-labeled anti-rabbit IgG was used as a secondary antibody to detect the primary antibody. The signals were measured using an enhanced chemiluminescence detection kit (Yeasen Biotechnology Co. Ltd. Shanghai, China).The following antibodies were used: c-KIT monoclonal antibodies (1:1000; Cell Signaling Technology, Boston, MA, USA), β-actin (1:1000; Beyotime Chemical Co.), anti-rabbit IgG, HRP-linked antibodies (1:1000; Cell Signaling Technology), anti-mouse IgG, HRP-linked antibodies (1:1000; Cell Signaling Technology), PDGFR-α monoclonal antibodies (1:1000; Cell Signaling Technology, United States), anti-TMEM16A (ANO1) antibodies (1:500; Abcam, Cambridge, MA, USA), anti-SK3 antibodies (1:500; ab192515; Abcam, Cambridge, MA, USA), and non-selective anti-PR antibodies (1:100; Newcastle upon Tyne, UK).

### 2.8 RNA extraction and real-time quantitative RT-PCR

Total ribonucleic acid (RNA) was extracted from smooth muscle tissue using a TRIzol reagent (TIANGEN, Beijing, China). RNA quality and quantity were examined using a NanoDrop 2000/2000c spectrophotometer (Thermo Fisher Scientific, Waltham, Massachusetts, USA). The mRNA was converted to cDNA using the Prime Script RT Reagent Kit with gDNA Eraser (Takara, Dalian, Liaoning, China) according to the manufacturer's protocol. Quantitative polymerase chain reaction (PCR) was conducted with specific primers by FastStart Universal SYBR Green Master Mix (Roche, Mannheim, Germany) on the 7500 Real Time PCR System (Applied Biosystems, Waltham, MA, USA). The expression of target genes relative to controls was analyzed by the ΔCT method. The sequences of the primers used were as follows:

Pdgfrα F-ATGACAGCAGGCAGGGCTTCAACG.

Pdgfrα R-CGGCACAGGTCACCACGATCGTTT

c-Kit F-CCTCATTGGCTTTGTGGTTGCAG

c-Kit R-ATGCGCCAAGCAGGTTCACAA.

Gapdh F-CCATGGAGAAGGCCGGGG.

Gapdh R-CAAAGTTGTCATGGATGACC.

### 2.9 Immunofluorescence

Colonic tissues were obtained as described above. Tissue samples were fixed with 4 % ice-cold paraformaldehyde for 8 h, dehydrated in 20 % sucrose, embedded as frozen tissue blocks and cut into 8–10 μm thickness frozen sections at −24 °C. Samples were incubated in 0.1 M phosphate-buffered saline (PBS) containing 10 % normal goat serum for 2 h at 24 °C to block non-specific binding and incubated with goat anti-PDGFRα/c-KIT/PR antibody (anti-PDGFRα, 1:200, AF1062, R&D systems, USA; anti-PR antibody, 1:100, Newcastle upon Tyne, UK; anti-*c*-KIT antibody, 1:200, Cell Signaling Technology, Boston, MA, USA) mixed with Triton-X100 (0.5 %, Sigma Aldrich, St. Louis, MO, USA) at 4 °C for 24 h. Samples were washed in 0.1 M PBS for 30 min, then incubated at 24 °C with cy3-conjugated anti-goat IgG (1:300; GB21404, Wuhan goodbio technology, China), Alexa Fluor 488-conjugated goat anti-rabbit IgG (1:100, Jackson Immuno Research, USA) and DAPI for 2 h. Images were acquired using a confocal laser-scanning microscope (Leica TCS SP8, Germany).

### 2.10 Statistical analysis

The data are presented as the means ± SE. The differences between groups were analyzed using one-way ANOVA, followed by Bonferroni's post-hoc test or the Student's unpaired *t*-test, if needed. Statistical significance was set at p < 0.05, and n-values correspond to the number of animals used in the indicated experiments.

## 3 Results

### 3.1 Changes in slow colon transit of pregnant mice compared with non-pregnant mice

**Fig. 1:**
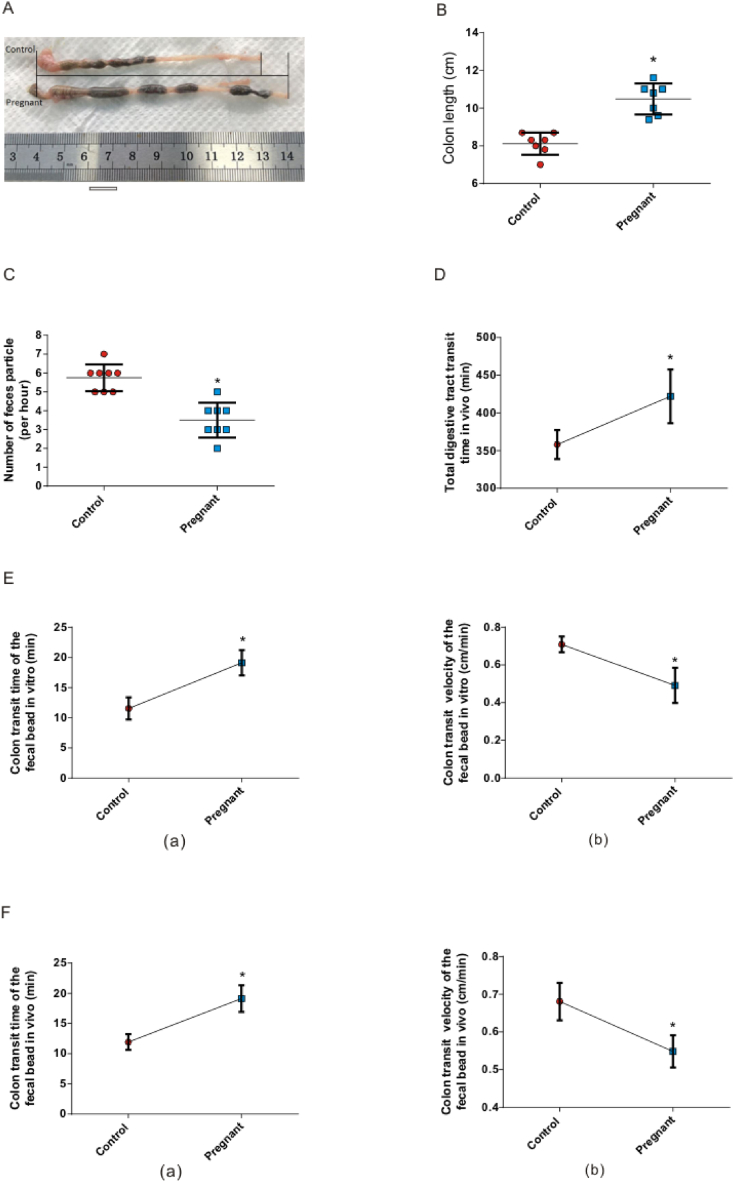
Changes in progesterone-induced colon slow transit of pregnant mice. A-B Morphology (A) and length (B) of colon in control group and pregnant mice group. C–D The number of defecation particles per hour (C) and the transit time of total digestion *in vivo* in mice (D). (E) The colon fecal-bead transit time (a) and velocity (b) of the control and pregnant mice *in vitro*. (F) The colon fecal-bead transit time (a) and velocity (b) of the control and pregnant mice *in vivo*. *, P < 0.05.

A comparison of the isolated colons between the pregnant mice and the control group (non-pregnant mice) showed that the length of the colons of pregnant mice increased significantly (Fig. 1A). The colon length of the control group was 8.11 ± 0.22 cm, while that of pregnant mice was 10.01 ± 0.43 cm (Fig. 1B; n = 7; P＜0.05). Next, we compared the amount of defecation per hour between the pregnant mice and the control group. We collected and counted the fecal granules of the mice for 24 h. The results showed that the number of fecal granules in pregnant mice was significantly decreased (Fig. 1C; n = 8; P < 0.05). Next, we compared the transit time of the whole digestive tract (from the stomach to the anus) *in vivo* between pregnant mice and the control group. The results showed that the transit time of the pregnant mice was significantly longer than that of the control mice (Fig. 1D; P < 0.05; n = 8). The transit time and speed in the colon were measured *in vitro*. The results showed that, compared with the control group, the colon fecal-bead transit time of pregnant mice *in vitro* was longer (Fig. 1E (a); P < 0.05; n = 8) and the transit speed was slower (Fig. 1E (b); P < 0.05; n = 8). Considering that the colon *in vitro* is separate from the internal environment *in vivo*, this may have affected the authenticity of the experiment. To further enhance the persuasiveness of the slow colon transit in pregnant mice, we also compared colon transit *in vivo* between pregnant and non-pregnant mice. The results further confirmed that the colonic transit of pregnant mice was slower than that of the control group (Fig. 1F (a); P < 0.05; n = 7), and the transit speed was slower (Fig. 1F (b); P < 0.05; n = 7).

### 3.2 Transit pattern of CMMC and the effect of progesterone on spontaneous colonic smooth muscle contraction

**Fig. 2:**
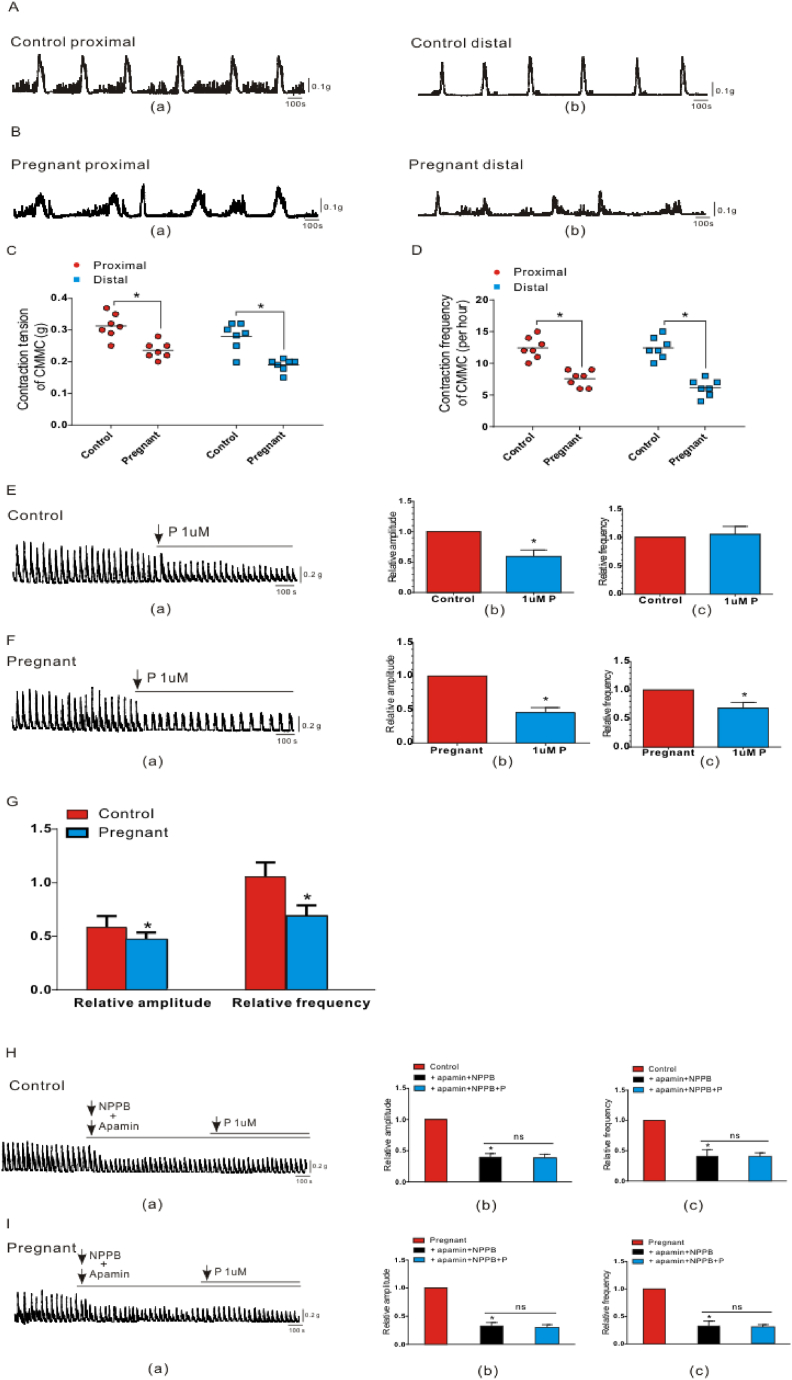
Transmission pattern of CMMC and the effect of progesterone on spontaneous contraction of the colonic smooth muscle. A-B Transmission waves of CMMC in the proximal and distal colon of control group (A) and pregnant mice (B). C–D Comparison of CMMC contraction tension (C) and frequency (D) between control and pregnant mice. E–F The effect of progesterone (E(a); F(a)) on the amplitude (E(b); F(b)) and frequency (E(c); F(c)) of spontaneous contraction of the colonic smooth muscle in the control group and pregnant mice group. G The difference in the effect of progesterone on spontaneous contraction of the colonic smooth muscle between the two groups. H–I In the presence of apamin and NPPB, the effect of progesterone (H(a); I(a)) on the amplitude (H(b); I(b)) and frequency (H(c); I(c)) of spontaneous contraction of the colonic smooth muscle in the control group and pregnant mice group. *, P < 0.05.

We first compared the transit patterns of CMMC in the colons of control and pregnant mice. When compared with the regular contraction amplitude and frequency of the control group (Fig. 2A (a)–(b)), the transit of CMMC in the colon of pregnant mice was irregular (Fig. 2B (a)–(b)), and the contraction frequency and amplitude were obviously slower than those of the control group (Fig. 2C–D; P < 0.05; n = 7).

To explore the effect of progesterone on colonic smooth muscle contractions, we performed isometric tension experiments in pregnant mice and controls. In both groups the amplitude decreased after the addition of 1 μM progestogen concentration and the contraction amplitude of pregnant mice decreased more obviously compared with before adding progesterone (Fig. 2E(a); (b); Fig. 2F(a); (b)). The contraction frequency of the control group remained the same as that at baseline before the addition of progesterone, but interestingly, the frequency decreased significantly in pregnant mice (Fig. 2E(a); (c); Fig. 2F(a); (c)). Compared to the control group, the contraction tension and frequency of colonic smooth muscles in pregnant mice were obviously weakened under the action of progesterone (Fig. 2G; P < 0.05; n = 8). These results show that the slow transit of the colon in pregnant mice may be related to the high levels of progesterone in their bodies.

In order to examine whether the inhibitory effect of progesterone on the contraction of colonic smooth muscle is caused by the regulation of PDGFRα^+^ cells and ICC, we first added apamin to block the SK3 channel on PDGFRα^+^ cells and NPPB to block the ANO1 channel on the ICC, and found that the contraction of colonic smooth muscle in both groups was obviously inhibited; while in the presence of apamin and NPPB, progesterone did not inhibit the contraction of smooth muscle in both groups (Fig. 2H and I; P < 0.05; n = 7). This result preliminarily shows that progesterone probably inhibits colon transit by regulating PDGFRα^+^ cells and ICC.

### 3.3 The effect of apamin and NPPB on progesterone-mediated inhibition of smooth muscle contraction

**Fig. 3:**
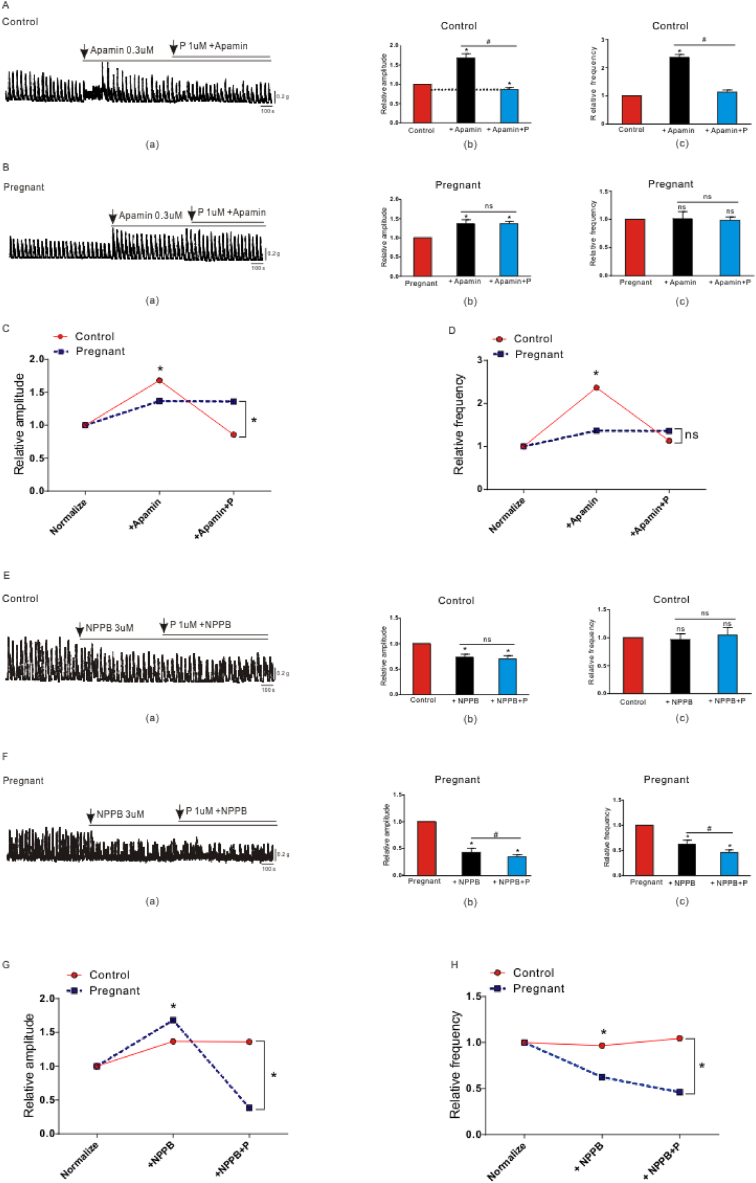
The effect of apamin and NPPB on progesterone-induced inhibition of smooth muscle contraction. A-B The effect of apamin and apamin + progesterone (A(a); B(a)) on the amplitude (A(b); B(b)) and frequency (A(c); B(c)) of spontaneous contraction in the control and pregnant mice group. C-D The difference in the effect of apamin and apamin + progesterone on the amplitude (C) and frequency (D) of spontaneous contraction between the two groups. E-F The effect of NPPB and NPPB + progesterone (E(a); F(a)) on the amplitude (E(b); F(b)) and frequency (E(c); F(c)) of spontaneous contraction in the control and pregnant mice group. G-H The difference in the effect of NPPB and NPPB + progesterone on the amplitude (G) and frequency (H) of spontaneous contraction between the two groups. *, P < 0.05.

Based on the above results, we then studied how progesterone regulates PDGFRα^+^ cells and ICC, and makes colon transit slow in pregnant mice. In the control groups, the amplitude and frequency increased in the presence of apamin, which blocked the SK3 channel (Fig. 3A(a)–(c)). In pregnant mice, the contraction amplitude increased in the presence of apamin, but the contraction frequency was not significantly different before and after the addition of apamin (Fig. 3B(a)–(c)). Progesterone was added when apamin blocked the SK3 channel, and we found that progesterone significantly inhibited the contraction frequency and amplitude of the control group, whereas the pregnant mouse group showed no obvious inhibitory effect (Fig. 3A(b); (c) and 3 B (b); (c)). In addition, compared with the pregnant mice group, the effect of progesterone in the control group was more obvious (Fig. 3C and D; n = 8; P < 0.05). Based on apamin blocking the relaxation of PDGFRα^+^ cells, the inhibitory effect of progesterone on pregnant mice was weaker than that of the control group, indicating that the contraction function mediated by the ICC was down-regulated in pregnant mice.

Next, we studied the regulation of progesterone on PDGFRα^+^ cells. In the control groups, the amplitude decreased in the presence of NPPB, which blocked the ANO1 channel, but the contraction frequency was not significantly different before and after the addition of NPPB (Fig. 3E(a)–(c)). In pregnant mice, the amplitude and frequency decreased in the presence of NPPB (Fig. 3F(a)–(c)). We then added progesterone when NPPB blocked the ANO1 channel in the ICC. We found that progesterone significantly inhibited the contraction frequency and amplitude in the pregnant mice group, while the non-pregnant control group showed no obvious inhibitory effect (Fig. 3E(b); (c) and 3F(b); (c)). In addition, compared with the control group, the effect of progesterone in the pregnant mice group was more obvious (Fig. 3G and H; n = 8; P < 0.05). Based on NPPB blocking the contraction of the ICC, the inhibitory effect of progesterone on pregnant mice was stronger than that of the control group, indicating that the relaxation function mediated by PDGFRα^+^ cells was up-regulated in pregnant mice.

### 3.4 The expressions of PR, c-KIT (ICC), ANO1, PDGFRα and SK3 in colonic smooth muscle tissue

**Fig. 4:**
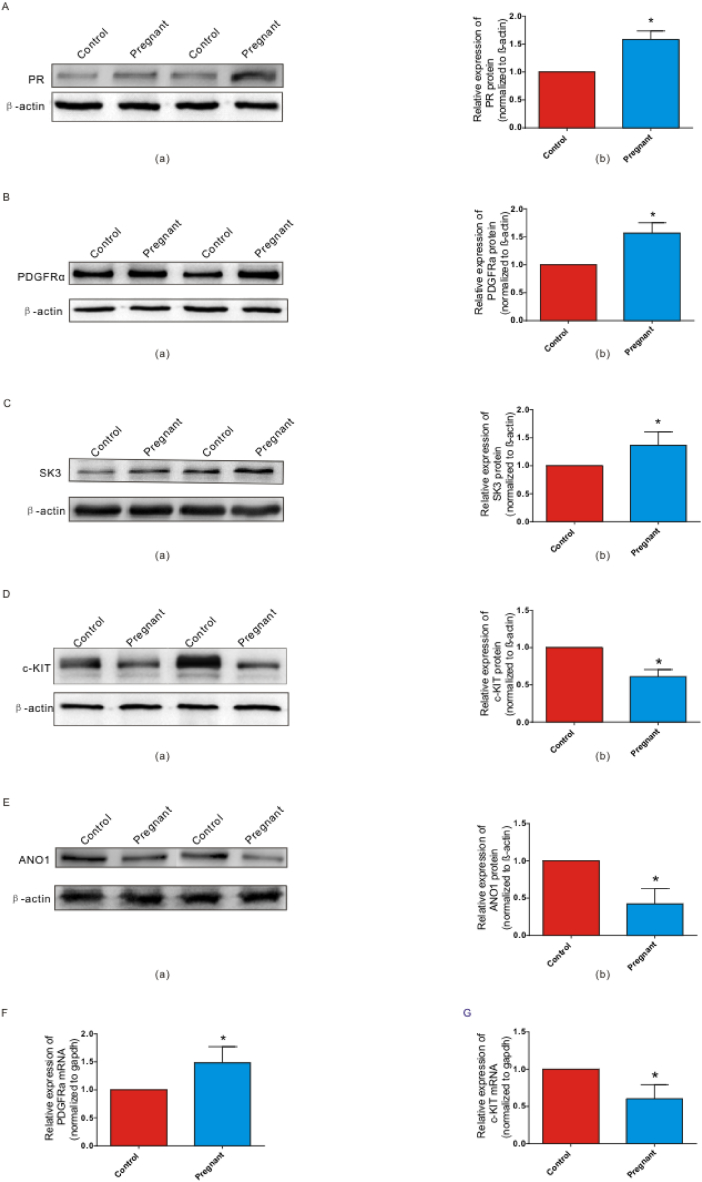
The expressions of PR, ICC (c-KIT), ANO1, PDGFRα, and SK3 in colonic smooth muscle tissues. A The protein level of PR (a) and gray intensity analysis (b) in the control and pregnant mice group. B–C The protein level of PDGFRα and SK3 (B(a); C(a)) and gray intensity analysis (B(b); C(b)) in the control and pregnant mice group. D–E The protein level of c-KIT and ANO1 (D(a); E(a)) and gray intensity analysis (D(b); E(b)) in the control and pregnant mice group. F–G The mRNA level of PDGFRα (F) and c-KIT (G) in the control and pregnant mice group.

Next, we performed Western blot and qRT-PCR to explore the mRNA and protein levels of progesterone, ICC, ANO1, PDGFRα, and SK3 in colonic smooth muscle tissue. The results showed that the protein expressions of PR, PDGFRα, and SK3 were significantly up-regulated in the pregnant mice group (Fig. 4A, B, and 4C), while the protein expressions of c-KIT and ANO1 were significantly down-regulated (Fig. 4D and E). These results suggest that the occurrence of colonic slow transit during pregnancy may be related to the changes in the regulation of PR on ICC and PDGFRα. The mRNA level of PDGFRα was obviously higher than those from the control group (Fig. 4F). In contrast, compared to the control group, the mRNA expression of c-KIT decreased significantly (Fig. 4G).

### 3.5 The localization of PR on PDGFRα^+^ cells and the ICC from colonic smooth muscle tissue

**Fig. 5:**
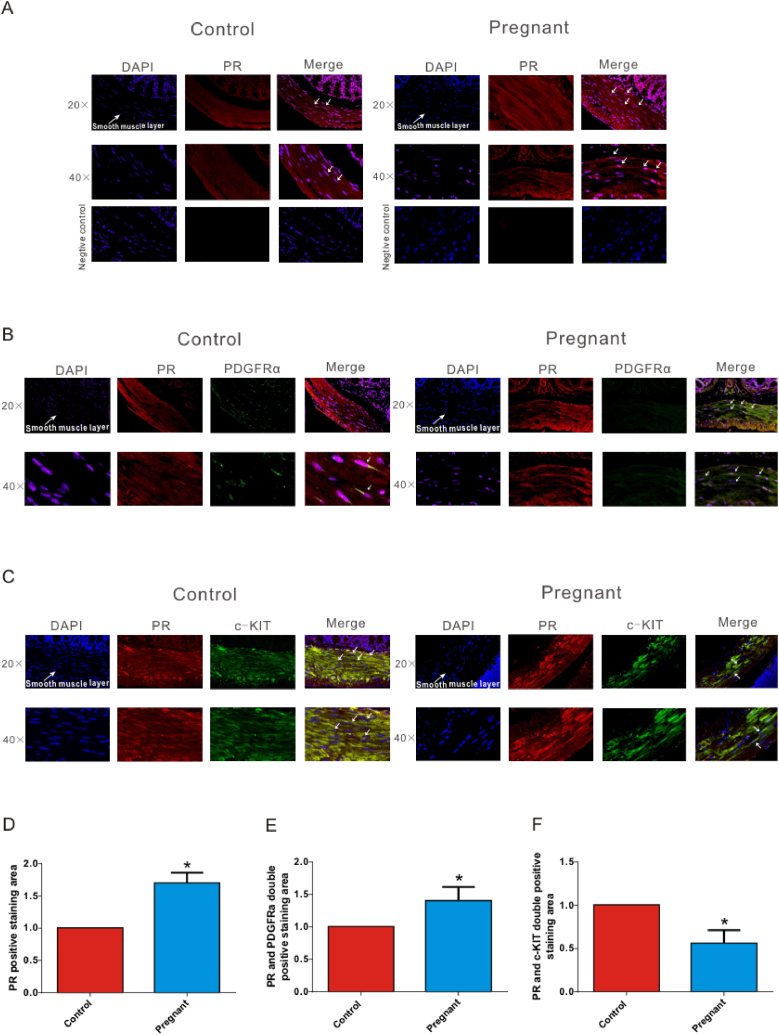
The localization of PR on PDGFRα^+^ cells and the ICC from colonic smooth muscle tissue. (A) Localization of PR in the colonic smooth muscle layer in the control and pregnant mice group. (B) The expression of PR co-located with PDGFRα^+^ cells in the smooth muscle layer from the control and pregnant mice group. (C) The expression of PR co-located with c-KIT (the identification of ICC) in the smooth muscle layer from the control and pregnant mice group. D The immunofluorescence positive staining area of PR in the control and pregnant mice group. E-F The immunofluorescence double positive staining area of PR/PDGFRα and PR/c-KIT in the control and pregnant mice group.

To explore the existence of PR on PDGFRα^+^ cells and the ICC, immunofluorescence was performed. First, we examined the distribution of PR in the colonic smooth muscle tissue. The results showed that PR was expressed in both groups and its expression in the colonic smooth muscle tissues of pregnant mice significantly increased. To quantify the expression of PR in smooth muscle layers, the positive staining area was measured and was found to be significantly increased using lasx software (Fig. 5A and D; n = 7; P < 0.05). Next, we explored the localization of the PR on PDGFRα^+^ cells and the ICC in the colonic smooth muscle layer. The results showed that PR localized stellate-shaped or spindle-shaped cells among the smooth muscle tissue, and the expression of PR co-located with PDGFRα^+^ cells from the pregnant mice group was more abundant than that in the control (Fig. 5B). In addition, compared to the control group, the expression of PR co-located with ICC decreased significantly (Fig. 5C). Similarly, we measured the double-positive staining area of PR/PDGFRα and PR/c-KIT by using lasx software, and found that the expression of PR co-located with PDGFRα+ cells was more (Fig. 5E; n = 5; P < 0.05) and the expression of PR co-located with ICC was less (Fig. 5F; n = 6; P < 0.05) in pregnant mice group compared with the non-pregnant control group.

## 4 Discussion

More than one-third of pregnant women regularly complain of bloating and constipation, particularly in the first and third trimesters [26]. However, owing to the peculiarity of pregnant women and the limitations of drug treatment, an increasing number of researchers are focusing on the mechanism of gastrointestinal transit disorders during pregnancy. Based on the existing literature, most researchers believe that an increased progesterone level has a great effect on gastrointestinal transit motility [[27], [28], [29], [30]]. However, most previous studies focused on the effect of progesterone on SMC, rather than ICC and PDGFRα^+^ cells that regulated the contractile and diastolic functions of smooth muscle [[31], [32], [33]]. To our knowledge, this is the first study of how progesterone decreases colonic transit during pregnancy by regulating PDGFRα^+^ cells and the ICC. Both *in vitro* and *in vivo* colon transit experiments confirmed slow transit of the colon in pregnant mice. We found that the amplitude and frequency of colonic smooth muscle contractions in pregnant mice were significantly inhibited compared to those in the non-pregnant control group. Fig. 2E and F shows that the addition of progesterone slowed colon transit in pregnant mice, indicating that pregnant mice expressed more endogenous PRs. We speculated that the increase of endogenous PRs may be related to the expression of more PDGFRα^+^ cells in the smooth muscle layers of pregnant mice. However, when apamin and NPPB block the function of PDGFRα^+^ cells and the ICC at the same time, the inhibitory effect of progesterone on colon transit is relieved, which further confirms progesterone inhibits colon transit by enhancing the relaxation of PDGFRα^+^ cells and weakening the contraction of ICC from the smooth muscle layers. Western blot and immunofluorescence experiments demonstrated that progesterone was more co-localized in PDGFRα^+^ cells and less co-localized in the ICC in the pregnant mice group, compared with the control group. These results confirm that under the two-way regulation of progesterone, the relaxation function of PDGFRα^+^ cell-SMC is enhanced, while the contractile function of ICC-SMC is weakened, ultimately leading to the slow colon transit of pregnant mice.

Previous studies concluded that pregnant women exhibit decreased colonic muscle activity, with progesterone playing a major inhibitory role. Kumar (1962) first found the inhibitory effect of progesterone on gastrointestinal activity [34]. Subsequently, Eliakim et al. examined the literature on the regulation of gastrointestinal motility from 1966 to 2000, but their findings were contradictory [35]. Progesterone plays a regulatory role in non-pregnant women. Preston et al. (1986, 1988) found that there were more female patients with gastrointestinal motility disorders than male patients, and that most female patients were significantly relieved during menstruation, which may be due to low progesterone levels during menstruation [36,37]. Therefore, most researchers believe that slow gastrointestinal transit during pregnancy is closely associated with increased progesterone levels. Up to now, although much literature reported that progesterone inhibited the contraction of gastrointestinal smooth muscle, no study explored the progesterone-mediated regulation of PDGFRα^+^ cells and the ICC in the gastrointestinal or colonic smooth muscle from animals or humans.

The PR, a member of the nuclear receptor superfamily, has two human receptor subtypes (PRA and PRB) in humans [38]. Researchers have demonstrated that these two protein receptors are structurally related but functionally different. In recent years, PRA has been reported to be mainly involved in regulating the physiological functions of the female reproductive system, such as the ovaries and uterus, whereas PRB mainly regulates functions outside the female reproductive system, such as the digestive and nervous system [39]. However, in this study, we used a nonselective anti-PR antibody. Therefore, whether progesterone plays a role in regulating colonic transit through the PRB subtype requires further investigation. A large number of experiments have shown that progesterone's nongenomic effects block Ca^2+^ release from intracellular stores without impairing Ca^2+^ influx and prove that progesterone affects intestinal smooth muscle cell contraction mainly through non-genomic pathways [32,40]. Female patients with slow-transit constipation have impaired colonic muscle contractions in response to agonists that act on G-protein-coupled receptors or stimulate G proteins directly [41]. Most studies have shown that progesterone inhibits intestinal smooth muscle cells mainly by increasing nitric oxide synthesis to induce smooth muscle relaxation. In addition, progesterone can inhibit signaling pathways that mediate contraction, such as Rho kinase [42,43]. Hermoso and Villalo found that interstitial ICC not only expresses connexin Cx43, but also that the level of sex hormones affect the expression and function of connexin [44,45]. Therefore, PR may affect ion channels and the level of intracellular Ca^2+^ in gastrointestinal smooth muscle cells through a non-genomic endocrine pathway and then regulate the contraction activity of gastrointestinal smooth muscle. Based on the above findings, we know that progesterone plays an obvious role in gastrointestinal function, and few studies have been conducted under pathological conditions, such as inflammatory bowel disease, pregnancy, and lactation. We aimed to explore the effect of SIP syncytium, as the motor unit of smooth muscle, which is composed of SMC, PDGFRα^+^ cells and the ICC through gap junctions, in progesterone-mediated slow colon transit in pregnant mice.

In conclusion, under the two-way regulation of increased progesterone, the relaxation function of PDGFRα^+^ cell–SMC is enhanced and the contraction function of ICC–SMC is weakened, leading to the slow colon transit of the pregnant mice. Our findings might have clinical implications by explaining the mechanism of the slow gastrointestinal transit diseases during pregnancy.

The current study has some limitations. The contraction function of the ICC is dominant in the proximal colon, while the relaxation function of PDGFRα^+^ cells is important in the distal colon, resulting in transit waves from near to far [46]. However, this study did not study the proximal and distal colon separately, so whether the regulation mechanism of progesterone on PDGFRα^+^ cells and the ICC in the proximal and distal colon is different remains unclear. In addition, we know that the smooth muscle layer not only has an SIP syncytium but also gap junctions between cells and develops the enteric nervous system (ENS) and various neurotransmitters secreted by the ENS [47]. Whether progesterone regulates PDGFRα^+^ cells and the ICC through gap junction proteins or neurotransmitters or even more complicated mechanisms is unknown. The underlying regulatory mechanism of progesterone on the ion current and membrane potential of ANO1 and SK3 channels still needs further exploration.

## Funding statement

This work was approved by the 10.13039/501100001809National Natural Science Foundation of China (82101769).

## Availability of data and materials

The data and materials are available from the corresponding author on reasonable requests.

## CRediT authorship contribution statement

**Chen Lu:** Writing – original draft, Project administration. **Hui Luo:** Data curation. **Ye Wang:** Formal analysis, Data curation. **Shuang Jing:** Methodology. **Jun Zhao:** Software. **Kexin Zou:** Data curation. **Fan Wu:** Data curation. **Hao Ying:** Writing – review & editing, Investigation, Data curation, Conceptualization.

## Declaration of competing interest

The authors declare the following financial interests/personal relationships which may be considered as potential competing interests:Chen Lu reports financial support was provided by 10.13039/501100001809National Natural Science Foundation of China. If there are other authors, they declare that they have no known competing financial interests or personal relationships that could have appeared to influence the work reported in this paper.
