# Supplementary material for: Regulation of PDGFRα+ cells and ICC in progesterone-mediated slow colon transit in pregnant mice
Source: Heliyon. 2024 Jan 28;10(3):e25227. doi: 10.1016/j.heliyon.2024.e25227 (PMC10850515; doi:10.1016/j.heliyon.2024.e25227)
Supplement: Multimedia component 1 [file mmc1.docx]

**PR**


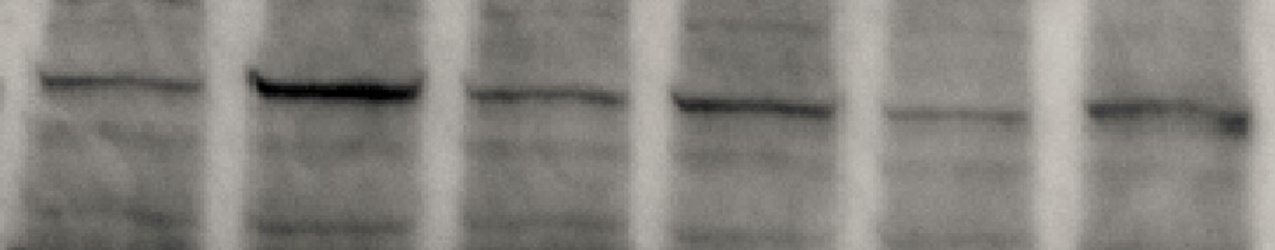


**β-actin**


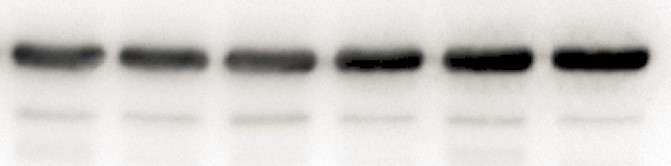


**Figure 4A(a)**

**PDGFRα**


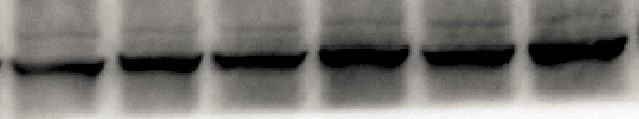


**β-actin**


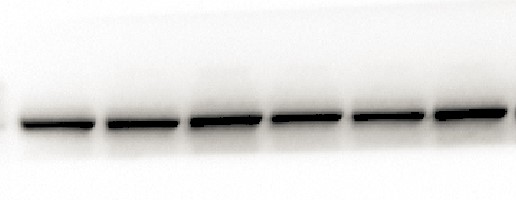


**Figure 4B(a)**

**SK3**


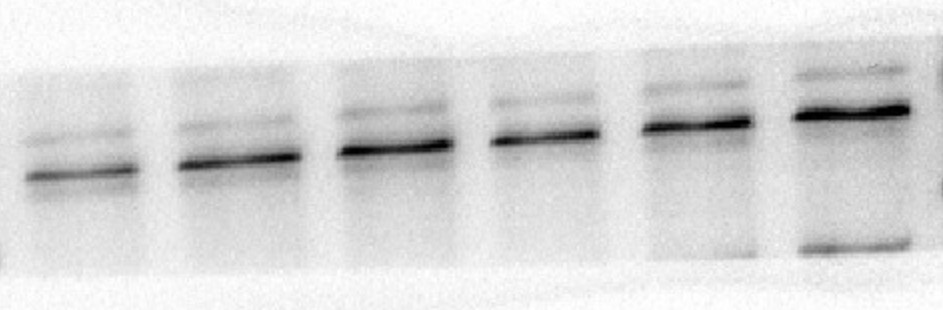


**β-actin**


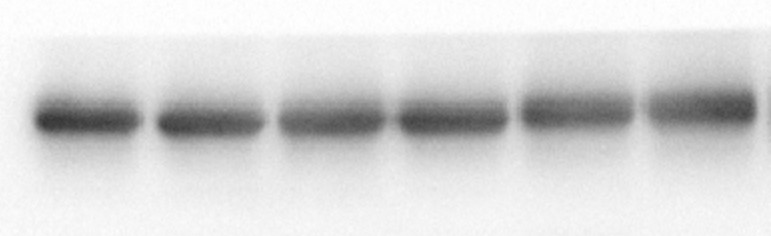


**Figure 4C(a)**

**c-KIT**


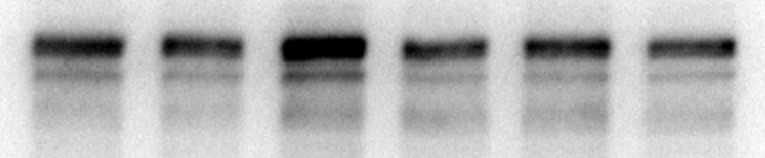


**β-actin**


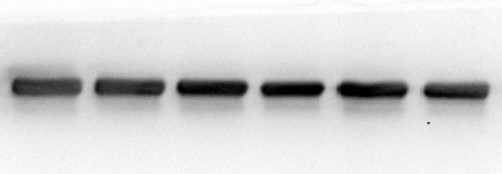


**Figure 4D(a)**

**ANO1**


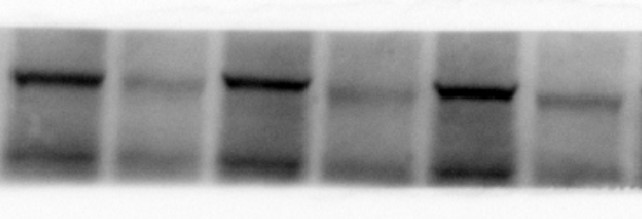


**β-actin**


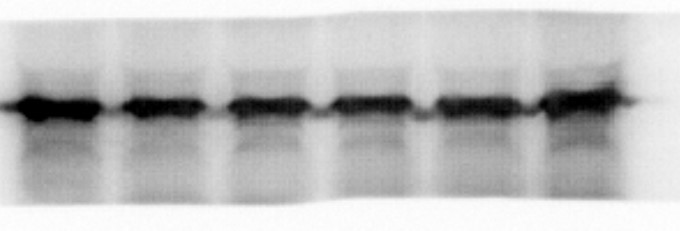


**Figure 4E(a)**
